# *Helicobacter Pylori* Infection in a Group of Egyptian Children With Upper Gastro-Intestinal Bleeding

**DOI:** 10.4021/gr533e

**Published:** 2013-07-14

**Authors:** Abdel-Azeem M. El-Mazary, Mostafa A. Elfoly, Magdy F. Ahmed, Waleed M. Abdel-Hamed, Zmzm M. Hassan

**Affiliations:** a Pediatric Department, Minia University, Minia city, Minia, Egypt; b Tropical-Medicine Department, Minia University, Minia city, Minia, Egypt; c Clinical-Pathology Department, Minia University, Minia city, Minia, Egypt

**Keywords:** *Helicobacter-pylori*, GIT bleeding, Variceal, Non-variceal bleeding

## Abstract

**Background:**

Upper gastrointestinal bleeding is a life threatening condition in children. Common sources of upper gastrointestinal bleeding in children include mucosal lesions and variceal hemorrhage. Helicobacter pylori (*H. pylori*) is a Gram negative spiral-shaped bacterium that is found in the gastric mucous layer or adherent to the epithelial lining of the stomach. It causes more than 90% of duodenal ulcers and up to 70-80% of gastric ulcers. The relationship between *H. pylori* infection and upper GIT bleeding in children is still un-clear. This study aimed to estimate the incidence of *H. pylori* infection in children presented with upper GIT bleeding and correlation between *H. pylori* infection and endoscopic findings of the cause of bleeding.

**Methods:**

The study included 70 children presented with upper GIT bleeding indicated for upper gastrointestinal endoscopy admitted in pediatric department, Minia University Hospital, Egypt during the period from February 2010 to December 2012. Thirty healthy children were included as a control group with age and sex matched. After medical history taking and physical examination all children were exposed for laboratory investigations (CBC, prothrombin time and concentration, liver function tests, hepatitis viral markers, blood urea and serum creatinine and *Helicobacter pylori* stool antigen test). Upper endoscopy was done for patients only. Patients were classified into variceal and non variceal groups according to upper endoscopy.

**Results:**

*Helico-pylori* infection was significantly higher in children with non-variceal bleeding than controls (P = 0.02) and children with variceal bleeding (P = 0.03) with no significant difference between children with variceal bleeding and controls (P = 0.9). Both weights and BMIs centile were significantly lower in variceal and non-variceal groups than controls (P = 0.01 & 0.001 and 0.01 & 0.001 respectively). AST, ALT and direct bilirubin levels were significantly higher in variceal group than controls (P = 0.001, 0.004 & 0.001 respectively). Prothrombin concentration and albumin levels were significantly lower in variceal group than controls (P = 0.001 & 0.01 respectively). Hemoglobin levels were significantly lower in variceal and non-variceal groups than controls (P = 0.01 & 0.001 respectively). No significant differences were present as regards age, sex, height or platelets count between cases (variceal and non-variceal) and controls.

**Conclusions:**

*H. pylori* infection is significantly higher in children with non-variceal bleeding than controls. No significant difference between children with variceal bleeding and controls. Triad of increased ALT, decreased albumin levels and negative *H. pylori* infection could be a significant triad in predicting variceal bleeding as a cause of upper GIT bleeding in children.

## Introduction

Upper gastrointestinal bleeding is a life threatening condition in children. Common sources of upper gastrointestinal bleeding in children include mucosal lesions and variceal hemorrhage [1, 2]. Incidence of upper GI bleeding was observed in 6.4-10% of pediatric ICU admissions [3, 4]. *Helicobacter pylori* (*H. pylori*) is a Gram negative spiral-shaped bacterium that is found in the gastric mucous layer or adherent to the epithelial lining of the stomach. *H. pylori* infection is related to more than 90% of duodenal ulcers and up to 70-80% of gastric ulcers [5-8]. In adults, the presence of *H. pylori* confers a six fold increased risk of gastric adenocarcinoma, accounts for half of all gastric cancers and strongly implicated in the development of gastric B cell mucosa associated lymphoid tissue (MALT) lymphomas [9]. Many studies investigated the relationship between *H. pylori* and upper GIT bleeding in adults [10-12], but no enough studies in children.

### Aim of the work

This study aimed to estimate the incidence of *H. pylori* infection in children presented with upper GIT bleeding and correlation between *H. pylori* infection and endoscopic findings of the cause of bleeding.

## Methods

This is a prospective study included 70 children presented with upper GIT bleeding indicated for upper gastrointestinal endoscopy admitted in pediatric department, Minia university hospital, Egypt during the period from February 2010 to December 2012. Thirty healthy children were included as a control group with age and sex matched. The study was approved by pediatric department council, Minia University and informed consents from parents or caretakers were taken. Children receiving any medications (NSAIDS, anticoagulants, corticosteroids) and those with known history of coagulopathy, bleeding disorders, diabetes mellitus or chronic illness were excluded from the study. All children after history taking and physical examination were exposed to laboratory investigations. Upper G.I.T endoscopy performed within 24 hours of admission for patients group only.

Blood samples were taken by sterile venipuncture: Two mls of venous blood on EDTA containing tube were aspirated for CBC. Another two mls were aspirated on sodium citrate containing tube for prothrombin time and concentration by thrombrel-s (human thromboplastin containing calcium) from (Behring diagnostic Inc. USA). Five mls of venous blood were aspirated on a plain plastic tube and left to be clotted in the incubator and centrifuged to be separated for assessment of liver function tests (using Integra 400 auto analyzer). Hepatitis B surface antigen was measured by Enzyme Linked Immunosorbent Assay (Sanofi Diagnostic Pasture, Marne-La-Coquette. France).Hepatitis C antibody was measured by a third generation ELISA (BIOELISA HCV Kit, BIOKIT, S. A Barcelona). Blood urea and serum creatinine were measured (by fully automated clinical chemistry auto-analyzer system Konelab 20i.). A fresh stool sample was collected and stored at -20 °C for analysis for *H. pylori* stool antigen test (Premier Platinum HpSA, Meridian Diagnostics, Cincinnati, OH) [13, 14]. Abdominal Ultrasound: done using a real time equipment (Fukuda Denshi - 4500) linear machine. Upper endoscopy was done for patients only. Olympus pediatric gastroscope (GIFP3) was used for the procedure [15].

### Statistical analysis

Data were analyzed using Statistical Package for the Social Science (SPSS for windows version 13.0). The continuous variables were expressed as mean ± SD which compared using chi-square test. Statistical significance was defined as a probability level of P ≤ 0.05. For calculation and comparison between weights and BMIs centiles we used Z-score. WHO centile charts were used for weight, height and BMI cantle measurements [16].

## Results

*Helicobacter pylori* infection was significantly higher in children with non-variceal bleeding than controls (P = 0.02) and variceal bleeding (P = 0.03) with no significant differences between children with variceal bleeding and controls (P = 0.9). All patients were negative for both hepatitis B and C markers. No significant differences between cases and controls as regards urea or creatinine levels were present. A laboratory triad of increased ALT (> 45 U/L), decreased albumin levels (< 4 g/dL) and negative *H. pylori* infection was a significant triad in predicting variceal bleeding as a cause in children presented with upper GIT bleeding. It was positive in 8 cases of 18 children presented with variceal bleeding (44.4%) versus only one case of 52 children presented with non-variceal bleeding (1.9 %) (P = 0.002). Non-Variceal group included: gastritis (38 patients), duodenitis (9 patients), esophagitis (2 patients), multiple duodenal polyps (1 patient) and Mallory Weiss tear (2 patients), (Table 1-3).

**Table 1:** Comparison Between Cases and Control Groups as Regards Demographic, Clinical and Laboratory Data

Item	Cases (no = 70)	Controls (no = 30)	P-value
Age (in years)			
Range	4 - 14	4 - 13	0.122
Mean ± SD	6.4 ± 3.23	5.3 ± 2.9	
Sex			
Male	44 (62.9%)	18 (60%)	0.8
Female	26 (37.1%)	12 (40%)	
Weight (centile)			
Range		5 - 75	0.041*
Mean ± SD	27 ± 19.2	56.5 ± 20.2	
Height (centile)			
Range	3 - 75	3 - 50	0.320
Mean ± SD	25.2 ± 16.4	48.7 ± 15.5	
BMI (centile)			
Range	3 - 75	50 - 90	0.001**
Mean ± SD	29.2 ± 22.9	51 ± 3.1	
Jaundice			
Yes	18 (25.7%)	0 (0%)	0.001**
No	52 (74.3%)	30 (100%)	
AST (U/L)			
Range	20 - 400	14 - 33	0.01*
Mean ± SD	51.3 ± 58.2	23.1 ± 5.5	
ALT (U/L)			
Range	10 - 387	10 - 30	0.04*
Mean ± SD	49.6 ± 61.2	20.8 ± 5.8	
Albumin (g/dL)			
Range	3 - 5.5	3.8-5.2	0.143
Mean ± SD	4.2 ± 0.8	4.3 ± 0.5	
P.C (%)			
Range	50 - 100	70 - 100	0.003**
Mean ± SD	81.5 ± 17.7	91.9 ± 8	
Total bilirubin (mg/dL)			
Range	0.3 - 9.5	0.4 - 1	0.006**
Mean ± SD	1.9 ± 2.4	0.7 ± 0.1	
Direct bilirubin (mg/dL)			
Range	0.1 - 7.3	0.1 - 0.4	0.014*
Mean ± SD	0.9 ± 1.6	0.1 ± 0.09	
Hemoglobin (g/dL)			
Range	7 - 13	11 - 15	0.001**
Mean ± SD	10.6 ± 1.3	12.8 ± 1.03	
Platelets (×10^3^/µL)			
Range	190 - 500	168 - 500	0.290
Mean ± SD	339.5 ± 106.2	314.7 ± 107.6	

*significant, **highly significant.

**Table 2:** Comparison Between Variceal and Control Groups as Regards Demographic, Clinical and Laboratory Data

Item	Variceal (no = 18) ^§^	Controls (no = 30)	P-value
Age (in years)			
Range	6 - 14	4 - 13	0.020*
Mean ± SD	8.6 ± 3.5	5.3 ± 2.9	
Sex			
Male	12 (66.7%)	18 (60%)	0.8
Female	6 (33.3%)	12 (40%)	
Weight (centile)			
Range	5 - 75	5 - 75	0.010*
Mean ± SD	21.7 ± 18.8	56.5 ± 20.2	
Height (centile)			
Range	3 - 50	5 - 90	0.130
Mean ± SD	22 ± 13.1	48.7 ± 15.5	
BMI (centile)			
Range	3 - 50	50 - 60	0.001**
Mean ± SD	21.7 ± 17.9	51 ± 3.1	
Jaundice			
Yes	14 (77.8%)	0 (0%)	0.006**
No	4 (22.2%)	30 (100%)	
AST (U/L)			
Range	34 - 400	14 - 33	0.001*
Mean ± SD	104.5 ± 92.2	23.1 ± 5.5	
ALT (U/L)			
Range	23 - 387	10 - 30	0.004*
Mean ± SD	111.3 ± 82.5	20.8 ± 5.8	
Albumin (g/dL)			
Range	3 - 4	3.8 - 5.2	0.01*
Mean ± SD	3.4 ± 0.3	4.3 ± 0.5	
P.C (%)			
Range	50 - 100	70 - 100	0.001**
Mean ± SD	64.4 ± 14	91.9 ± 8	
Total bilirubin (mg/dL)			
Range	0.8 - 9.5	0.4 - 1	0.001**
Mean ± SD	4.2 ± 2.7	0.7 ± 0.1	
Direct bilirubin (mg/dL)			
Range	0.1 - 7.3	0.1 - 0.4	0.001**
Mean ± SD	2.5 ± 2.1	0.1 ± 0.09	
Hemoglobin (g/dL)			
Range	7 - 11	11 - 15	0.01**
Mean ± SD	9.4 ± 0.8	12.8 ± 1.03	
Platelets (×10^3^/µL)			
Range	190 - 500	168 - 500	0.731
Mean ± SD	303 ± 117.7	314.7 ± 107.6	

§Variceal group includes: gastric varices (3 patients) and esophageal varices (15 patients), *significant, **highly significant.

**Table 3:** Comparison Between Non-Variceal and Control Groups as Regards Demographic, Clinical and Laboratory Data

Item	Non-Variceal (no = 52) ^§^	Controls (no = 30)	P-value
Age (in years)			
Range	4 - 10	4 - 13	0.348
Mean ± SD	6.01 ± 3.3	5.3 ± 2.9	
Sex			
Male	32 (61.5%)	18 (60%)	0.9
Female	20 (38.5%)	12 (40%)	
Weight (centile)			
Range	3 - 75	5 - 75	0.01*
Mean ± SD	29.9 ± 17.5	56.5 ± 20.2	
Height (centile)			
Range	5 - 75	3 - 50	0.532
Mean ± SD	26.3 ± 17.4	28.7 ± 15.5	
BMI (centile)			
Range	3 - 75	50 - 90	0.001**
Mean ± SD	31.7 ± 24.07	51 ± 3.1	
Jaundice			
Yes	4 (7.7%)	0 (0%)	0.3
No	48 (92.3%)	30 (100%)	
AST (U/L)			
Range	20 - 79	14 - 33	0.09
Mean ± SD	37.1 ± 12.2	23.1 ± 5.5	
ALT (U/L)			
Range	10 - 55	10 - 30	0.08
Mean ± SD	24.7 ± 9.3	20.8 ± 5.8	
Albumin (g/dL)			
Range	3 - 5.5	3.8-5.2	0.803
Mean ± SD	4.4 ± 0.6	4.3 ± 0.5	
P.C (%)			
Range	50 - 100	70 - 100	0.122
Mean ± SD	87.3 ± 14.8	91.9 ± 8	
Total bilirubin (mg/dL)			
Range	0.3 - 0.8	0.4 - 1	0.179
Mean ± SD	1.5 ± 1.1	0.7 ± 0.1	
Direct bilirubin (mg/dL)			
Range	0.1 - 3.5	0.1 - 0.4	0.242
Mean ± SD	0.6 ± 0.3	0.1 ± 0.09	
Hemoglobin (g/dL)			
Range	8 - 13	11 - 15	0.001**
Mean ± SD	11.3 ± 1.01	12.8 ± 1.03	
Platelets (×10^3^/µL)			
Range	190 - 500	168 - 500	0.118
Mean ± SD	352.03 ± 100.1	314.7 ± 107.6	

§Non-variceal group includes: gastritis (38 patients), duodenitis (9 patients), esophagitis (2 patients), multiple duodenal polyps (1 patient) and Mallory Weiss tear (2 patients), *significant, **highly significant.

## Discussion

*H. pylori* was shown to be associated with peptic ulcer disease in 1985. Since then, the detection of spiral bacterium in the gastric mucosa has become a principal aspect of the diagnosis of patients with upper gastrointestinal symptoms suggestive of peptic ulcer disease [6, 8].

It is now generally accepted that *H. pylori* infections are acquired during childhood or adolescence in developing as well as developed countries with prevalence in children ranged from less than 10% to greater than 80% dependening upon age, socioeco­nomic class and geographic distribution [17, 18].

In this study, *H. pylori* infection was significantly higher in children with non-variceal bleeding than control group (65.4% vs. 36.7% P = 0.02) and variceal group (65.4% vs. 33.3% P = 0.03) with no significant difference between children with variceal bleeding and control group (33.3% vs. 36.7% P = 0.9). This is partly in agreement with the previous as well as other studies [10, 17, 18].

These results may be due to the strong positive correlation between *H. pylori* and both of gastric and duodenal ulcers which represented the main causes of bleeding in our study.

Hsiao et al, 2011 [10] and Gisbert et al, 2007 [12] reported that *H. pylori* eradication was associated with a reduced risk of ulcer recurrence and/or bleeding and these results supports our findings.

The higher rate of infection with *H. pylori* and the associated gastritis in those children are alarming signs, as infection at a young age is believed to result in chronic atrophic gastritis and gastric cancer in adult [9] which deserve immediate action.

In this study no significant difference between males and females was present and these results were in agreement with other reports [17-19], while Leandro et al, 2005 [20] reported a significant association between *H. pylori* infection and male gender (without explanation).

Ages of children presented with variceal bleeding ranged from six to fourteen years (with mean age 8.6 ± 3.5 years). This may be due to the progression in the severity of varices over time [20, 21].

Both weights and BMIs centile were significantly lower in variceal and non-variceal groups than controls (P = 0.01 & 0.001 and 0.01 & 0.001 respectively). These results reflected the bad nutritional aspect of those children either due to chronic liver disease or *helicobacter-pylori* infection. These results were comparable to other studies [18-20].

Abnormal liver function tests in the form of elevated ALT and AST, decreased prothrombin concentration, prolonged prothrombin time, elevated bilirubin levels, decreased albumin and total protein levels in children of variceal bleeding may be due to presence of chronic liver disease or due to hepatic hypo perfusion secondary to upper GIT bleeding.

In this study hemoglobin levels were lower -as expected- in cases (variceal and non-variceal) than controls, since our cases presented by upper GI bleeding and most of them were *H. pylori* positive and this is in agreement with other studies reported association between *H. pylori* infection and presence of anemia [21-23].

*H. pylori* infection may cause iron deficiency anemia through sequestration of iron by antral *H. pylori* infection, malabsorption of iron, folic acid, vitamin B6 and vitamin B12 [23, 24] and\or by an autoimmune process triggered by antigenic mimicry between *H. pylori* epitopes and major autoantigens of the gastric mucosa [25].

An etiology scoring system was published by Pongprasobchai et al, in March 2009 in the world journal of gastroenterology for predicting the etiology of upper GI bleeding in adults depending upon both clinical and laboratory data [26], but no scoring system developed in children yet.

The results of this study revealed that a laboratory triad of increased ALT levels (> 45 U/L), decreased albumin levels (< 4 g/dL) and negative *H. pylori* infection was a significant triad in predicting variceal bleeding as a cause in children presented with upper GIT bleeding as this triad was positive in 9 children, 8 of them (88.8%) were variceal bleeding in origin.

This laboratory triad reflected the un-compensated condition of the liver for those children having variceal bleeding, this is not in accordance with a study [27] reported that 76.5% of children with gastrointestinal bleeding in north India were patients of extrahepatic portal vein obstruction. This difference may be attributed to the high incidence of Bilharsiasis and its complications in Egypt responsible for liver affection and portal hypertension.

No significant differences were found as regards demographic data between H. pylori-positive and H. pylori-negative children except for age where older children had a higher rate of infection. This indicates that the prevalence curve of acquisition of *H. pylori* infections rises with age, which may be due to outdoor activities and exposure to potential external sources. These results were in agreement with other studies [19, 20].

No significant difference between cases (variceal and non variceal groups) and controls was present as regards platelets count and this is not in agreement with other studies reported association between *H. pylori* infection and idiopathic thrombocytopenic purpura [28, 29].This difference is attributed to our exclusion criteria as we excluded children with known history of coagulapathy or bleeding disorder from this study.

### Conclusion

*H. pylori* infection is significantly higher in children with non-variceal bleeding than controls. No significant difference between children with variceal bleeding and controls. Triad of increased ALT, decreased albumin levels and negative *H. pylori* infection could be a significant triad in predicting variceal bleeding as a cause of upper GIT bleeding in children.
